# Neuronal correlates of eyeblinks are an expression of primary consciousness phenomena

**DOI:** 10.1038/s41598-023-39500-z

**Published:** 2023-08-03

**Authors:** Alejandro Luis Callara, Alberto Greco, Enzo Pasquale Scilingo, Luca Bonfiglio

**Affiliations:** 1 Dipartimento di Ingegneria dell’Informazione, University of Pisa, Via G. Caruso 16, 56122 Pisa, Italy; 2 Research Center “E. Piaggio”, University of Pisa, Largo Lucio Lazzarino 1, 56122 Pisa, Italy; 3 Department of Translational Research and New Technologies in Medicine and Surgery, University of Pisa, Pisa, Italy; 4 Unit of Developmental Neurorehabilitation, Maternal and Child Department, Pisa University Hospital, Pisa, Italy

**Keywords:** Biomedical engineering, Cognitive neuroscience

## Abstract

The blinking rate far exceeds that required for moistening the cornea and changes depending on whether a person is resting or engaged in cognitive tasks. During ecological cognitive tasks (such as speaking, reading, and watching videos), blinks occur at breakpoints of attention suggesting a role in information segmentation, but the close relationship between cognition dynamics and blink timing still escapes a full understanding. The aim of the present study is to seek (1) if there is a temporal relationship between blink events and the consecutive steps of cognitive processing, and (2) if blink timing and the intensity of blink-related EEG responses are affected by task-relevance of stimuli. Our results show that, in a classical visual oddball task, (i) the occurrence of blinks is influenced by stimuli, irrespective of their relevance, (ii) blinks following relevant stimuli are only apparently delayed due to the need of finalizing a behavioural response, and (iii) stimulus relevance does not affect the intensity of the blink-related EEG response. This evidence reinforce the idea that blinks are not emitted until the last step of the processing sequence has been completed and suggests that blink-related EEG responses are generated by primary consciousness phenomena which are considered by their nature non-modulable (all-or-nothing) phenomena.

## Introduction

The frequency of spontaneous blinking (Blink Rate, BR) at rest averages around 12 blinks per minute in humans^1^, showing a wide interindividual variability influenced by multiple factors such as age, gender, personality, and vigilance^2–4^. Blink’s role is not limited to preventing tear film break up, for which 3 blinks per minute would be sufficient, but has been found to be involved in other functions^5,6^.

### Spontaneous blinking and visual continuity

Spontaneous blinks go generally unnoticed by the subject who performs them. In fact, despite eyelids closure causes about 100 ms interruptions of incoming visual information (a period that would be distinctly perceived if corresponding to a break of the illumination), our subjective experience is that of a continuous and stable vision (visual continuity). This is attributed, at present, to three alternative or complementary phenomena: the visual cortex suppression induced by either a corollary discharge to the motor command that generates the blink^7–9^ or by the sensory proprioceptive consequences of eyelid closure^10^, and the memory representation of the latest image seen before eyelids closure so as to fill in the perceptual gap^11–14^. It is interesting to note how the phenomenon of visual continuity, even if through partially different mechanisms, also occurs across rapid shifts of gaze (saccades) to reduce retinal blur^15^.

### Spontaneous blinking and dopamine-driven cognition

Spontaneous BR is closely associated with central dopaminergic (DA) function, in the sense that a high BR indicates increased DA activity, and a low one indicates reduced DA activity. This has been documented for several central nervous system diseases, where a BR increase corresponds to DA hyperactivity states (e.g., schizophrenia) and a BR reduction to DA deficiency states (e.g., Parkinson's disease)^2^, and confirmed by neuropharmacological studies, where DA agonists increase BR and DA antagonists reduce it^2^. The BR, therefore, can be considered an indirect marker of central DA function and, as such, can also be used to investigate the individual DA-related cognitive performance, mainly in relation with reward behaviour^16,17^ and executive (cognitive) control^4^. In fact, DA-driven cognition is the result of DA-dependent regulation of the decision threshold for both selecting responses (reward behaviour) and updating representations (executive control)^18,19^.

As far as the reward behaviour is concerned, reinforcement learning is governed by a balance between positive reinforcement (i.e., results better than expected; reward) -triggering a D1-mediated Go learning signal-, and negative reinforcement (i.e., results worse than expected; punishment) -triggering a D2-mediated NoGo learning signal^2^. A low BR, in particular, predicts learning from negative outcomes^16,17^, while a high BR is associated with the effort people are willing to spend on rewarded behaviour^20^, and then with their motivated behaviour and sense of agency^21^.

Regarding the executive control, it is classically described as the result of a dynamic balance between cognitive stability (i.e., the maintenance of task-relevant cortical representations in the face of task-irrelevant distractors, or gate closing), which is supported by D1 receptors in the prefrontal cortex, and cognitive flexibility (i.e., the updating of such representations in response to changing situational requests, or gate opening), which is driven by D2 receptors in the striatum^18,19^. In this context, a high BR predicts an increased ability to update representations related to new input information and, conversely, a low BR predicts an increased ability to maintain such representations^18,19^. However, the relationship between BR and actual performance does not follow a linear relationship, but rather an inverted-U shaped profile, that is the performance is compromised at two extremes of the curve, which correspond to situations of distractibility (high DA activity, high BR) and perseveration (low DA activity, low BR), respectively^2,3^. Moreover, BR undergoes temporal variations during the different phases of a visual working memory (WM) scanning-task showing a positive correlation with performance during the delay period, as a result of the balance between maintenance and updating of representations, and a negative correlation with encoding and probe periods, that are more related to visual attention^22^. Interestingly, a BR increase has also been shown in association with both updating and gate switching phases (either shifting to an updating mode or to a maintenance mode) of a WM reference-back task^23^ (see “Discussion” Section below). Nevertheless, a too high BR may be associated with poor performance in a WM N-back task, representing high levels of distractibility in the face of the task-dependent need to maintain cognitive stability^24^.

### Spontaneous blinking and ecological cognitive activities

The BR changes depending on cognitive activities the subject has to cope with, not only for laboratory experimental tasks, but also for more ecological situations such as reading, speaking or viewing videos^25–30^. The temporal distribution of blinking is not regular but varies and adapts according to the different moments of the current cognitive activity^14^. For example, activities requiring the engagement of visual attention lead to a global BR reduction^27,30–32^ so as to minimize information loss due to untimely blinks with respect to incoming information^33^. Nevertheless, the occurring blinks tend to cluster at attentional breakpoints (i.e., explicit or implicit pauses of the information flow) where this risk is minimal^27,30–32^. This is also true for speaking, when blinks tend to concentrate during the natural pauses between sentences^31,32^.

Thus, spontaneous blinking has been proposed as a dynamic mechanism through which the brain segments the stream of incoming information^34^ according to task intrinsic processing demands, in a similar way to what happens for saccades^35^. In this perspective, therefore, two opposing functional phases can be identified: blink inhibition, i.e. the time interval between two successive blinks (inter-blink interval, IBI), during which attention is focused on information processing^36^; and blink facilitation, during which an attentional shift takes place^37^ and information acquired in the previous phase is short term stored^27,38,39^. It was hypothesized that the inhibitory phase lengthens when more attentional resources are allocated, while shortens when voluntary attention fades due to fatigue or habituation^14^. The IBI, therefore, could also be defined as an attentional span, i.e. the time period in which attention is somehow active in relation to the changing environmental demands^14^.

Moreover, the blink, being located between two contiguous attentional spans, might represent the behavioural equivalent of an attentional set shifting, which in turn will be explicit (i.e., blinks associated with saccades) if directed toward another visual stimulus^35^, or implicit (i.e., blinks without saccades) if exerted upon the same visual stimulus -so renewing its perception^40^- or directed to inner thoughts^13^. In support of this, converging evidence has been recently accumulated about a transient blink-related activation of the precuneus (PCu) both in human^41–43^ and non-human primates^10^. Precuneus is one of the main hubs of the default-mode network (DMN)^44^, which is known to be activated during resting periods and linked to intrinsic, self-referential mental activity^45^. On the contrary, its extrinsic counterpart, the task-positive network (TPN), is activated during goal-directed cognitive tasks and linked to task-related mental activity^46^. It was hypothesized that, at the blink, a momentary switch occurs between TPN and DMN, corresponding to a flip of the attentional focus orientation from the external to the internal environment^37^. Why this happens has not yet been fully clarified. However, considering that PCu is involved in self-processing events with a visuospatial connotation^47^ such as gathering information from the surroundings and representing the self in relationship with the outside world^45,48^, blinking at rest has been hypothesized to operate as an automated surveillance mechanism of the visuospatial environment dealing with basic visuospatial awareness^41^ without the subject having to cope with any cognitive task. Interestingly, on the other hand, a mental arithmetic subtraction task, while increasing BR as an index of cognitive load, has been shown to decrease the blink-related activation of the precuneus^49^ due to competition on the same cortical area.

### Spontaneous blinking and blink-related EEG analysis to extract BRPs

Blink-related potentials (BRPs) can be analyzed as classical event-related potentials (ERPs) by time-locking the EEG traces on the moment in which a complete eyelid closure (T0) occurs, and which corresponds to the apex of the greatest deflection on the electrooculogram (EOG) or the corresponding frontopolar EEG-derived signal (see for example^50^). However, the blink is not an instantaneous event, but lasts, depending on the studies, between 200 and 400 ms^12^. According to Volkmann^7^, about 100 ms of its duration correspond to the lowering phase of the eyelids, which culminates with the achievement of complete closure, and the remainder to the reopening phase. Therefore, the eyelids closing phase precedes T0 and the opening phase follows it, while the flow of visual information is completely interrupted for a period of about 100 ms corresponding to the complete coverage of the pupil by eyelids.

Notoriously, each blink produces a spurious signal on the EEG, the blink artifact, not directly generated by brain's bioelectrical activity variations but produced by an external event to the brain (i.e., the change in the electrical potential of the corneo-retinal electric dipole caused by the eyelids passage over the cornea)^51^. This artifact, of great amplitude (especially on the frontal regions), covers for its entire duration the true EEG signal. This is the reason why, in most event-related potentials (ERPs) studies, EEG epochs contaminated by such an artifact are removed from the analysis. On the contrary, to enable the analysis of the authentic EEG modifications connected to the blink, the blink artifact must be removed by keeping intact the true underlying EEG signal^13,43^. After that, EEG epochs, aligned at time T0, can undergo time–frequency analysis (blink-related spectral perturbations, BRSPs) or, as in the present study, the extraction of the corresponding BRPs. It is important to stress that in the present work, to facilitate the understanding of both aims and results of our study, we chose to use the term BRPs for reasons of analogy towards ERPs, but it has to be considered as synonym of blink-related oscillations (BROs), term by which they are best known in literature.

BRPs are characterized by a bi- or triphasic waveform where the positive component peaks at about 300 ms from T0^13,43,52,53^. Due to their spatiotemporal features and to the simultaneous desynchronization of alpha oscillations, BRPs are considered similar to the P300 component of event-related potentials (ERPs)^13,14^ and, as such, are thought to have similar context updating and (short-term) memorization meanings (see^54^). According to this interpretation, the current environment image appearing at the eyelid reopening would be compared with its mnestic representation stored in the short-term memory at eyelid closure. In this way, despite the blink-dependent interruption of vision, it would be possible to check the match/mismatch of the two images and, thus, for adaptive reasons, to obtain information about the stability/instability of the environmental conditions^14^.

### Aims and scope

Due to their poor temporal solutions, parameters such as average BR and average IBI would not seem appropriate to investigate the intimate relationships between blink and cognition during ongoing goal-oriented behaviour. In fact, although they allow a comparison between tasks, these parameters do not faithfully reflect the cognition dynamics that occur within tasks. Accordingly, some authors have recently proposed the measurement of instantaneous blink rate^36,55^ to analyze blink-related cognitive processing from a trial-to-trial perspective^2,56^.

In the same vein, relating single blinking acts with the events of a classic experimental test, such as the oddball paradigm used in the present study, would seem a more appropriate approach than only using average BR and IBI. In such classical experimental test, in fact, events are very well differentiated from each other and accurately timed so as to constitute real temporal anchor points upon which to perform event-related analyses.

In the present work, we investigated whether cognitive task conditions modulate blinking behaviour and its neuronal correlates. In particular, we aimed at understanding whether eye blinking implies primary consciousness phenomena related to the visual-spatial context or more complex phenomena such as cognitive stimulus-processing. To do this, we analysed an open EEG data set collected by Walz and colleagues^57^ during a standard visual oddball task. Compared to Walz’s study, we introduced the main novelty of broadening the analysis towards blinking behaviour and bioelectrical brain activity related to the blink. Specifically, we analysed the event-related potentials by time-locking EEG epochs either to stimuli or behavioural motor responses or blinks themselves^13,14,41,58^ in order to unveil specific components related to the event of interest and by avoiding them being hidden by the background bioelectrical brain activity. This allowed us to study, in addition to the event-related potentials (ERPs) that partially reproduced the results of Walz's study, also the blink-related potentials (BRPs) that represented the main novelty of our work.

To the best of our knowledge, the present study was the first one that combined together the analyses of the temporal distribution of blinks related to stimuli presentation, the standard ERPs and the BRPs to investigate the blinking behaviour and the neural correlates of both cognitive stimulus-processing and blinking, during a visual oddball task.

Particularly, we investigate whether (1) the timing of the blink is influenced by the timing of the stimuli and/or the behavioural response; (2) the blink-related neural activity is influenced by the stimulus relevance; and, finally, (3) there is a relationship between stimulus-locked potentials and blinks emitted during goal-directed behaviour.

## Materials and methods

### Ethics statement

The study does not involve direct human participants, thus informed consent was not required. Fully detailed description of the dataset can be found in^57,59,60^. All methods were carried out in accordance with relevant guidelines and regulations of the Columbia University Institutional Review Board^59^.

### Dataset description

The dataset comprises EEG recordings from 17 subjects performing a visual oddball and an auditory oddball task. Here, we used only the data coming from the visual oddball since the auditory counterpart was performed in a closed-eyes condition^60^. Briefly, the experimental protocol consisted of 3 runs of a visual oddball task, each of which was made of 125 stimuli: 100 stimuli (i.e., the 80%) were standard (STND) stimuli to be ignored and 25 (i.e., the 20%) were target (TRGT) stimuli requiring a behavioural motor response (button press). Stimuli were administered for 200 ms with an inter-trial-interval (ITI) uniformly distributed between 2 and 3 s. Additionally, the first 2 stimuli were constrained to be standards.

Among the shared datasets (i.e., raw data, gradient-free data, re-referenced gradient-free data), we exploited the re-referenced gradient-free EEG data. According to^59^, these datasets are obtained after preprocessing the raw data (sampling frequency = 1 kHz, 43-channel custom cap with bipolar derivations) as follows: (i) high-pass filtering at 1 Hz to remove DC drift, (ii) notch-filtering at 60 Hz and 120 Hz for removing the line noise component and its first harmonic, and low-pass filtering at 100 Hz to remove high-frequency artifacts not associated with neurophysiological processes, (iv) EEG referencing from 43 bipolar channels to the 34-electrode space allowing for proper scalp topography analysis of EEG.

#### ICA decomposition and component selection

EEG signals measured on the scalp were decomposed into sets of (maximally-) statistically independent components with ICA^61,62^. For each subject, we estimated the number of components equal to the rank of the data, calculated as the non-zero eigenvalues of the eigen decomposition of the covariance matrix of EEG data at a tolerance of 1e−10. Such components represented brain activity as well as different types of artifacts (muscular, ocular, and other sources of noise). ICs were pre-tagged in each dataset as brain ICs or artefacts with a semiautomatic procedure involving the ICLabel EEGLAB plugin^63^, and by visual inspection (see “Blink related activity identification” Section for more details on component labeling procedure).

Then, components were clustered at the group level with the procedure presented in^64^. Briefly, the clustering vector is made of subject-specific mixing matrices (i.e., the spatial ICA maps) and the clustering procedure is constrained so that each subject can contribute to a cluster with at most one component. This method allows for controlling the false-positive rate (FPR) of the detected clusters of components, and the false discovery rate (FDR) of joining components to the clusters, determining which components are reliable (significant) enough in a statistically principled way^64^. Moreover, this procedure was found to be particularly suited to cluster ICs when the number of electrodes is limited^65^. Accordingly, we estimated clusters by controlling both FDR and FPR with a statistical threshold of α = 0.05.

The average map of each cluster was then described by means of an equivalent current dipole. Briefly, we used the boundary element model of the head based on the template from Montreal Neurological Institute (MNI; Montreal, Canada) and estimated each dipole with a two-stage procedure. First, we designed a grid of 34 equally spaced points ranging from − 85 to + 85 mm in the X and Y direction, and of 17 points ranging from 0 to + 85 mm in the Z direction. Then, among all the grid points, we restricted dipoles reconstruction to only grey matter positions based on a segmentation of the MNI template^66^. Then, nonlinear optimization of dipole position and orientation was performed by minimizing the error between the model and the measured potential^67^. Finally, we used EEGLAB^68^ to tag each dipole as the nearest area of grey matter according to the Desikan-Killiany atlas^69^.

### Blink related activity identification

The ICA performed on EEG signals was further exploited to extract useful information about blinking activity. Particularly, we aimed at identifying the peak of each blink to study the EEG responses locked to blink acts (i.e., to extract the BRPs). First, we classified ICs using the ICLabel EEGLAB plugin^63^. Then, we visually inspected ICs for identifying potentially misclassified components (e.g., eye components classified as non-eye components or viceversa). Among eye ICs, we identified blinks ICs based on their scalp maps and time courses. The time courses of subject-specific blink-related ICs were further analyzed with MATLAB custom scripts for identifying the peak of each blink (MATLAB. (2020). version 9.9.0.1857802 (R2020b). Natick, Massachusetts: The MathWorks Inc.). Accordingly, we could obtain for each subject blink specific peaks (hereinafter called blink times—BT). These BT were used as time-locking points for extracting grand average BRPs. Particularly, we considered only the first blink in the (0,1500)ms time-window following the stimulus administration, while excluding subsequent blinks in the same window.

### Grand Average ERP extraction, alignment, and comparison

Different grand average ERPs were obtained based on different time-locking events of interest. Particularly, we extracted three types of epochs: stimulus-locked epochs (SL), response-locked epochs (RL) and blink-locked epochs (BL) by synchronizing the EEG signals to stimulus administration, response times and blink times, respectively. Then, based on the type of the administered stimulus, epochs were further divided into STND (i.e., standard) and TRGT (i.e., target). Accordingly, we obtained five types of epochs: SL-STND, SL-TRGT, RL-TRGT, BL-STND, and BL-TRGT. Of note, RL epochs were available only for TRGT epochs as STND did not require any response. SL-STND and SL-TRGT epochs were extracted from − 500 to + 1500 ms around stimulus administration with 0 ms corresponding to the stimulus administration. Analogously, RL-TRGT epochs were extracted from − 500 to + 1500 ms around the subject’s specific response times with 0 ms corresponding to the instant of subject-specific responses. Instead, BL-STND and BL-TRGT were extracted from − 1500 to + 500 ms around the subject’s specific BT with 0 ms corresponding to the subject’s specific blink peaks. For BL epochs, we considered only the first blink in the (0,1500) ms time window following the stimulus administration, while excluding subsequent blinks in the same window. This different epoch extraction for BL epochs guarantees that even BL epochs with relatively late blinks do not overlap with the subsequent epoch while maintaining a satisfactory overlap with the SL epochs. For each type of epoch, we removed a subtractive baseline estimated in the 500 ms preceding the stimulus administration. Finally, grand average ERPs were obtained for each type of epoch.

Grand average ERPs were analyzed by performing the following comparisons:

1. SL-STND versus SL-TRGT
2. SL-STND versus RL-TRGT
3. SL-TRGT versus RL-TRGT
4. SL-STND versus BL-STND
5. SL-TRGT versus BL-TRGT
6. BL-STND versus BL-TRGT

For each comparison, we estimated significant differences among ERPs at the group level by means of paired statistics (t-tests), and by further controlling multiple hypothesis testing through the False-Discovery-Rate correction method^70,71^. Differences were estimated for all the time points following stimulus administration for comparisons 1–5, and for all the time points following the blink peak for comparison 6.

The motivation for studying epochs locked to different events is to reduce the temporal dispersion (i.e., jitter) of late ERP responses. Indeed, by aligning EEG epochs to each event in the cascade of events from the stimulus administration to the blink act one allows highlighting ERP components arising from each specific event (i.e., stimulus, response, blink)^41,72^.

Comparisons (1), (2) and (3) were performed to resemble the results of^59^ and thus to confirm the correctness of the adopted processing pipeline. Conversely, comparisons (4), (5) and (6) represent the main contribution of this work and are performed to highlight BRPs (comparisons 4 and 5) as well as to verify a possible influence of stimulus intrinsic cognitive load on the BRP amplitude (comparison 6).

Finally, for visualization purposes, we aligned the two grand average ERPs by selecting specific time points in the epoch. SL-STND versus SL-TRGT were aligned between them by matching the time point of stimulus administration (i.e., 0 ms). Instead, SL-STND versus RL-TRGT and SL-TRGT versus RL-TRGT were aligned by matching the time point of SL epochs corresponding to average response latency (i.e., 397.2 ms) with the 0 ms time point in the RL epochs (i.e., response times). In a similar fashion, SL-STND versus BL-STND and SL-TRGT versus BL-TRGT were visualized by aligning the time point of SL epochs corresponding to the average blink latency with the 0 ms timepoint in the BL epochs (i.e., blink times 595 ms for STND vs 779 ms TRGT). Finally, BL-STND versus BL-TRGT were visualized by aligning the time points corresponding to 0 ms in each epoch.

#### Windows of interest

**Figure 1:**
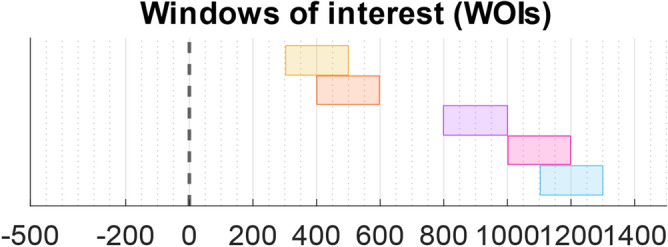
Windows of interest (WOIs). Dashed-line (0 ms): stimulus administration. Yellow WOI: 300–500 ms. Orange WOI: 400–600 ms. Purple WOI: 800–1000 ms. Pink WOI: 1000–1200 ms. Cyan WOI: 1100–1300 ms.

Although the statistical analyses were performed for each time point after the stimulus administration in the SL and RL epochs, and for each time point after the blink peak in the BL epochs, we focused our interpretation on some prespecified windows of interest (WOIs). More specifically, to facilitate the assessment of the statistical results obtained, we focused on WOIs where differences were expected on the basis of the instant of alignment of the epochs (Fig. 1) and on previous findings about visual P300 and BRPs^13,41,59,72,73^.

The WOIs were the following, in increasing temporal order:

1. From 300 to 500 ms, focusing on ERPs from both SL-STND and SL-TRGT, and corresponding to the expected latency of the P300 component (Yellow WOI);
2. From 400 to 600 ms, focusing on ERPs from RL-TRGT, and corresponding to the expected latency of the P300 response-locked components (Orange WOI);
3. From 800 to 1000 ms, focusing on BRP for SL-STND, which was obtained by adding the average blink latency from STND (i.e., 595 ms) plus the expected latency of BRP from blink (i.e., 300 ms) (Purple WOI);
4. From 1000 to 1200 ms, focusing on BRP for SL-TRGT, which was obtained by adding the average blink latency from TRGT (i.e., 779 ms) plus the expected latency of BRP from blink (i.e., 300 ms) (Pink WOI);
5. From 1100 to 1300 ms, focusing on BRP for RL-TRGT, which was obtained by adding the average latency of the motor response from TRGT (i.e., 397 ms) plus the average blink latency from motor response (i.e., 511 ms) plus the expected latency of BRP from blink (i.e., 300 ms) (Cyan WOI).
6. Additionally, we considered a WOI from 200 to 400 ms (i.e., around the expected latency of BRP from blink), focusing on BRP from blink-locked BL-STND and BL-TRGT which is not reported in Fig. 1.

#### Region and cluster of interest

We focused on event-related responses at the Pz electrode, with the only contribution of clusters localised in the precuneus based on several previous studies on blink-related oscillations^41–43,58,73^ and on robust anatomo-topographical relationships between Pz and precuneus^74^. Accordingly, the activity captured by other ICs not belonging to these clusters was excluded from the analysis (e.g., eye, muscle and other type of ICs).

## Results

The epoched data were satisfactorily clean, resulting in a total of 300 SL-STND and 75 SL-TRGT epochs per subject. Additionally, as reported in^59^, all subjects responded with high accuracy and speed to the target stimuli. The 98.4% ± 3.1% of targets were correctly detected, with 397.2 ± 38.9 ms response time (RT)^59^. Accordingly, RL-TRGT epochs were on average 74 ± 1 (median ± median absolute deviation -mad-) per subject. Concerning BL analysis, we observed that 46% of STND epochs and 48% of TRGT epochs had blinks in the (0,1500) ms window following stimulus administration. Accordingly, we obtained an average of 138 ± 51 (median ± mad) BL-STND epochs and 37 ± 12 (median ± mad) (BL-TRGT epochs.

### Independent component selection

**Figure 2:**
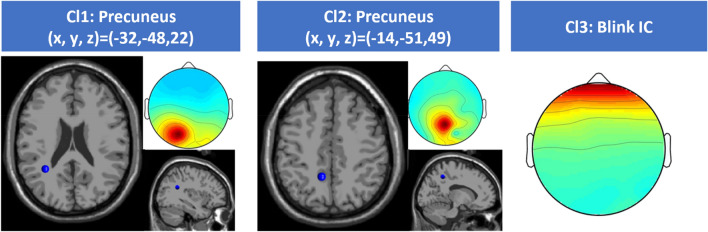
Cl1 and Cl2: cluster centroids of Brain ICs used to reconstruct the EEG signal on the scalp. For each cluster, the average IC map and its associated equivalent current dipole on the MNI standard space are reported. Cl3: average IC map of blink IC centroid.

Among the total, 5 clusters included contributions from multiple runs and multiple subjects and were associated with brain activity. Among these, two were described by a current dipole located in the precuneus as expected from the aforementioned previous studies. Accordingly, we reconstructed the EEG signal on the scalp by using only the contribution of the components joining these two clusters (Fig. 2). Moreover, we isolated a blink cluster (Blink IC) whose centroid is reported in Fig. 2 and whose time-courses were used to analyse the blinking activity.

#### Temporal distribution of blinks

**Figure 3:**
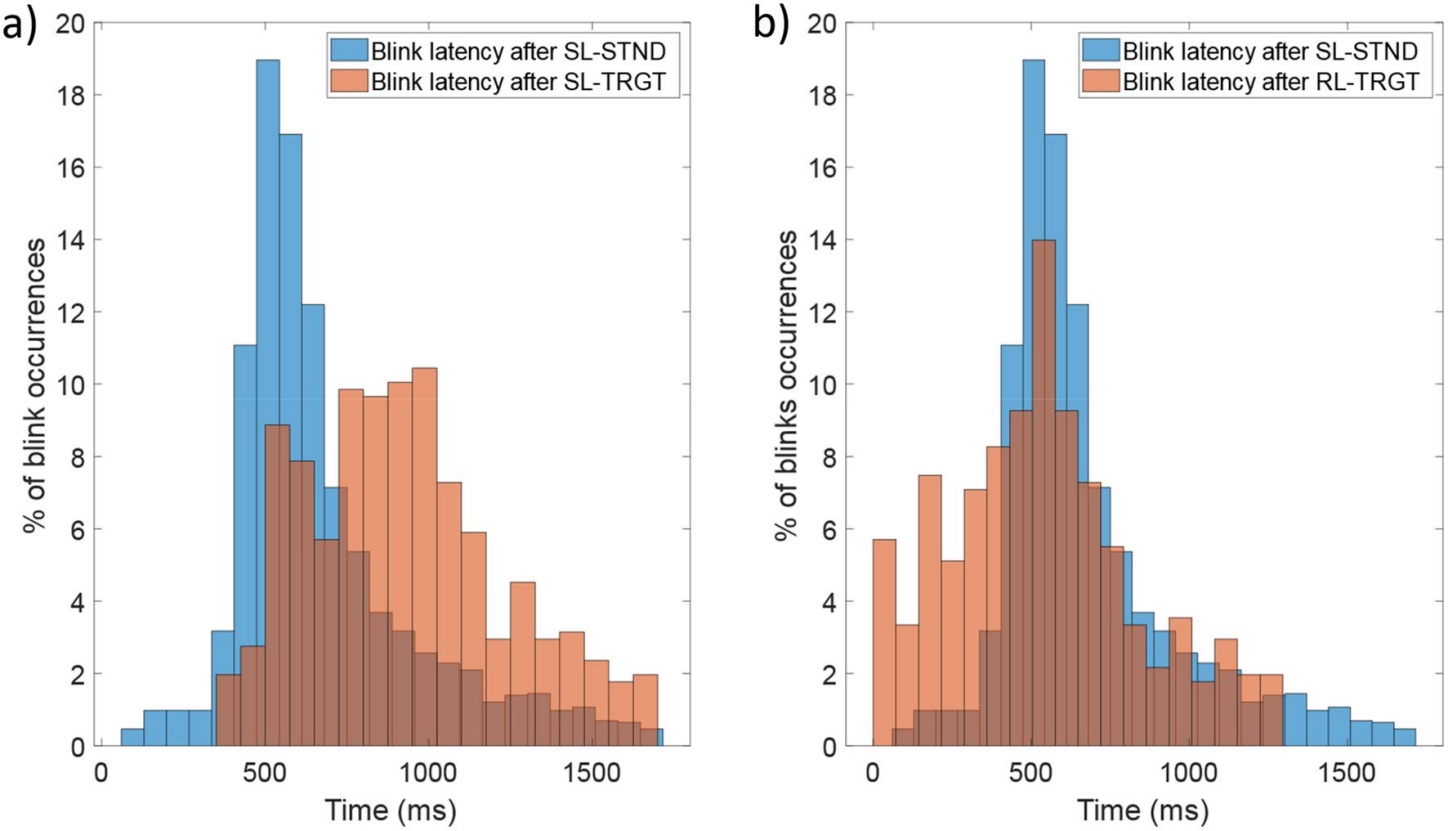
(**a**) Distribution histogram of blink latencies for SL-STND (mean blink latency: 595 ms) and SL-TRGT (mean blink latency: 779 ms) epochs. (**b**) Distribution histogram of blink latencies for SL-STND and RL-TRGT (mean blink latency: 511 ms from mean RT) epochs.

**Figure 4:**
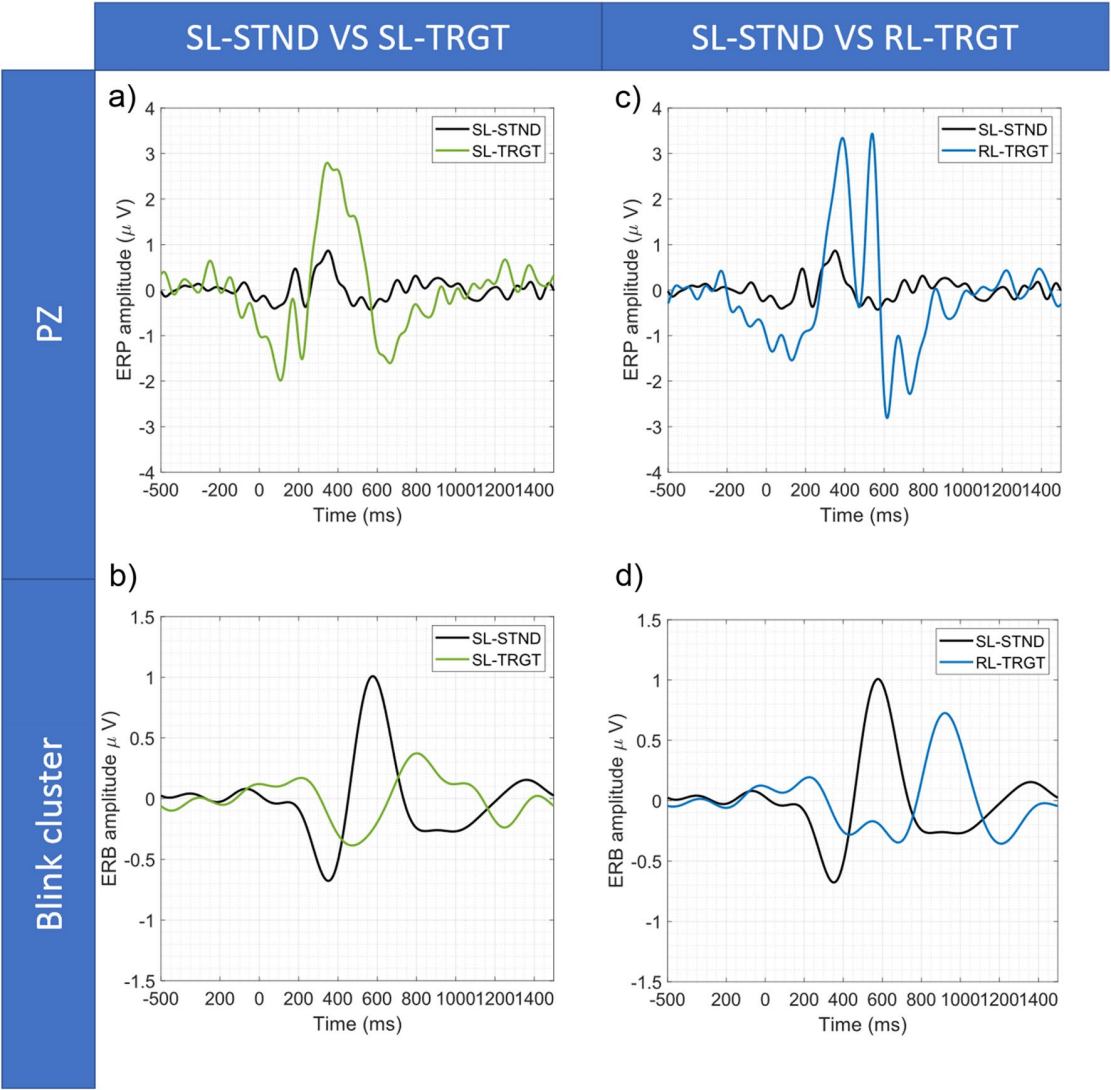
(**a**) SL-STND versus SL-TRGT ERPs at Pz Electrode. (**b**) SL-STND versus SL-TRGT ERPs of blink-cluster (i.e., Event-Related-Blink, ERB). (**c**) SL-STND versus RL-TRGT ERPs at Pz electrode. (**d**) SL-STND versus RL-TRGT ERPs of blink cluster (i.e., Event-Related-Blink, ERB).

The temporal distribution of blinks (Fig. 3a,b) suggests that, although blinks do not occur in response to all delivered stimuli but only to a portion of them (46% of standards and 48% of targets), they are driven either by stimuli or motor responses (Fig. 4b,d). In particular, blinks following target stimuli occur later (mean blink latency: 779 ms) than those following standard stimuli (mean blink latency: 595 ms) (Fig. 3a). However, if we align blinks to the button press rather than to the target stimulus, the resulting mean blink latency (511 ms) is comparable to that of the blink following the standard stimulus (Fig. 3b). This suggests that the latency of the blink from what we can consider the last meaningful event (i.e., the standard stimulus administration or the motor response to the target stimulus) remains substantially unchanged.

Moreover, in Fig. 4, we report the grand-average ERP and the grand-average event-related blink (ERB) for SL-STND versus SL-TRGT (Fig. 4a,b) and for SL-STND versus RL-TRGT (Fig. 4c,d), respectively. The average ERB peaked about 200 ms after the P300 component, showing that eyeblinks occur after the closure of the stimulus processing epochs and after the behavioural motor response (Fig. 3a,b).

#### Comparisons between SL-, RL-, and BL- event-related potentials

**Figure 5:**
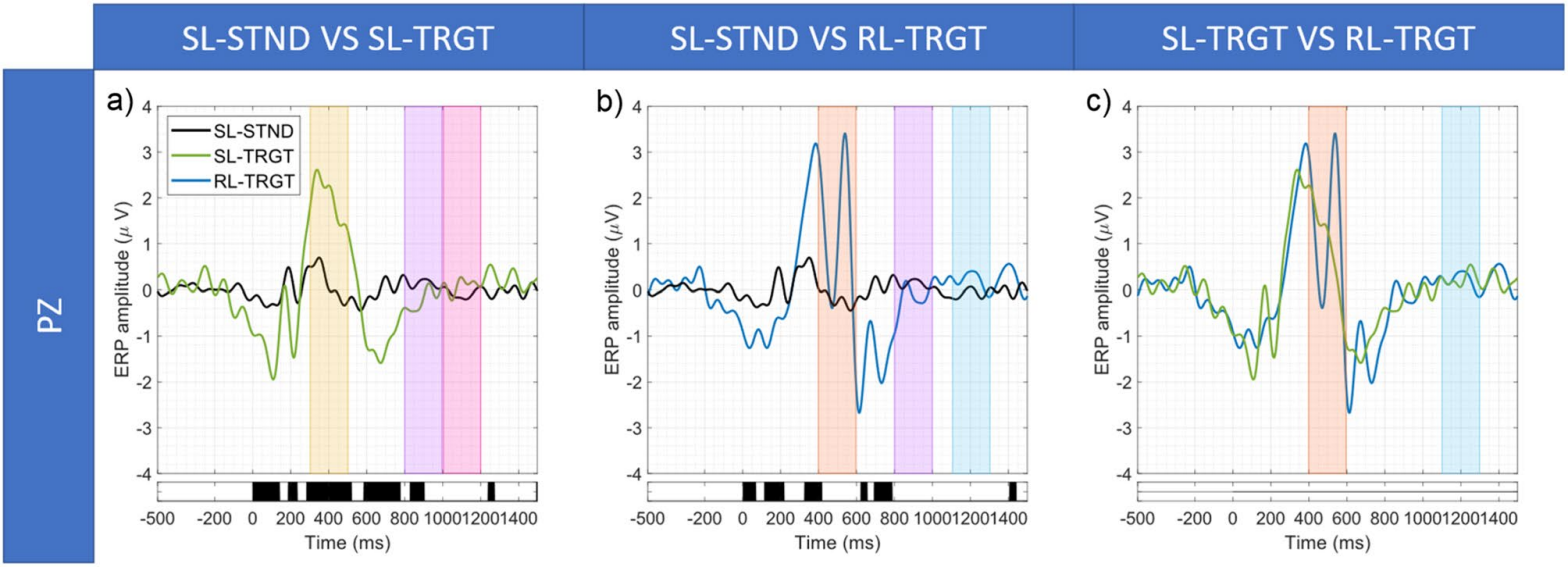
(**a**) SL-STND versus SL-TRGT. (**b**) SL-STND versus RL-TRGT. (**c**) SL-TRGT versus RL-TRGT. Grand avearge ERPs along with expected WOIs. For each comparison, black bars below the plot indicate the time-ranges for which differences between ERPs were statistically significant (*p* < 0.05, FDR-corrected).

**Figure 6:**
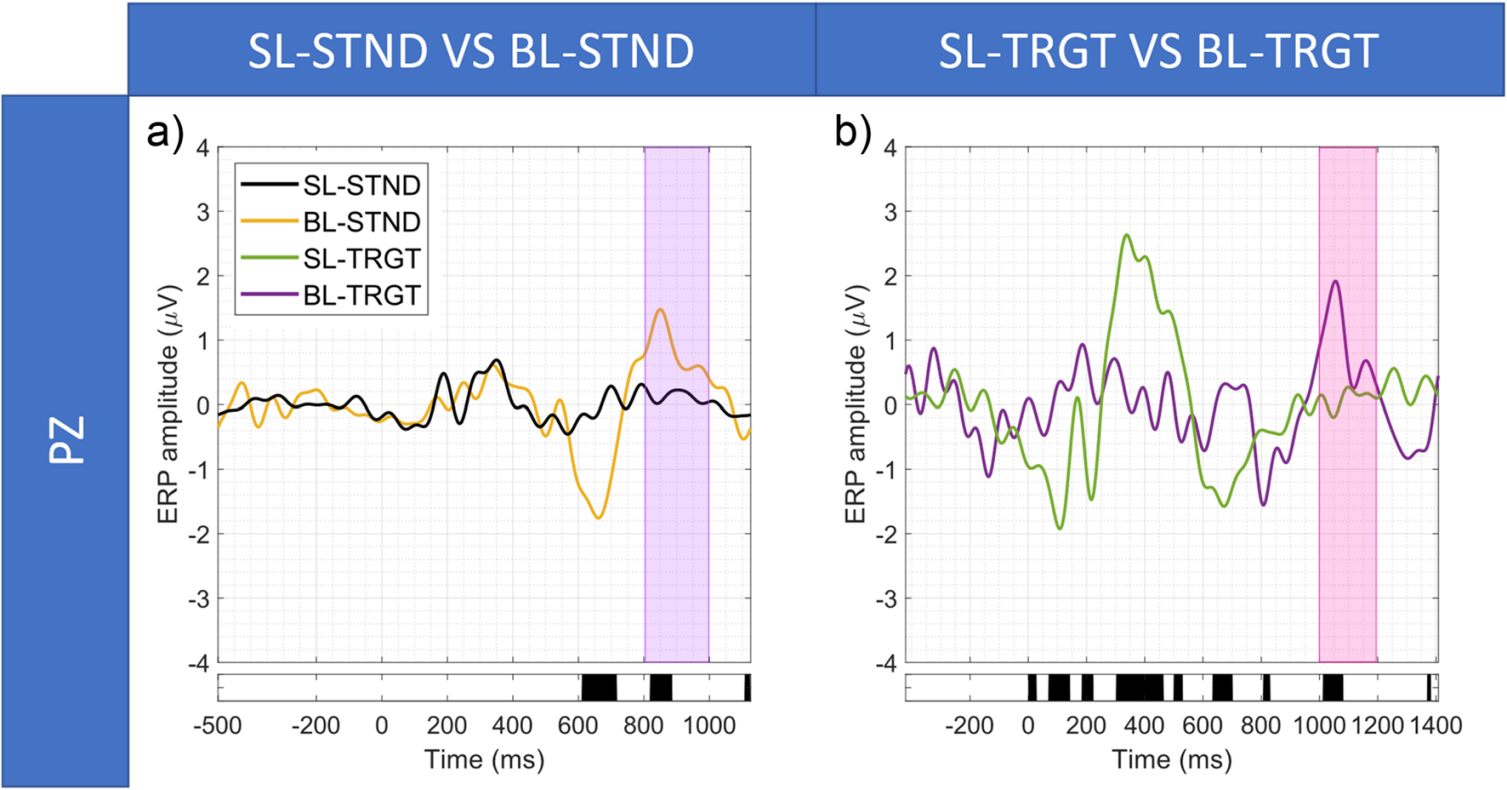
(**a**) SL-STND versus BL-STND. (**b**) SL-TRGT versus BL-TRGT. Grand average ERPs along with expected WOIs. For each comparison, black bars below the plot indicate the time-ranges for which differences between ERPs were statistically significant (*p* < 0.05, FDR-corrected). SL-STND versus BL-STND and SL-TRGT versus BL-TRGT were aligned by matching the 0 ms time point of BL ERPs with the average blink latency of the BL on the SL ERP time axis (i.e., 595 ms for SL-STND vs BL-STND and 779 ms for SL-TRGT vs BL-TRGT).

**Figure 7:**
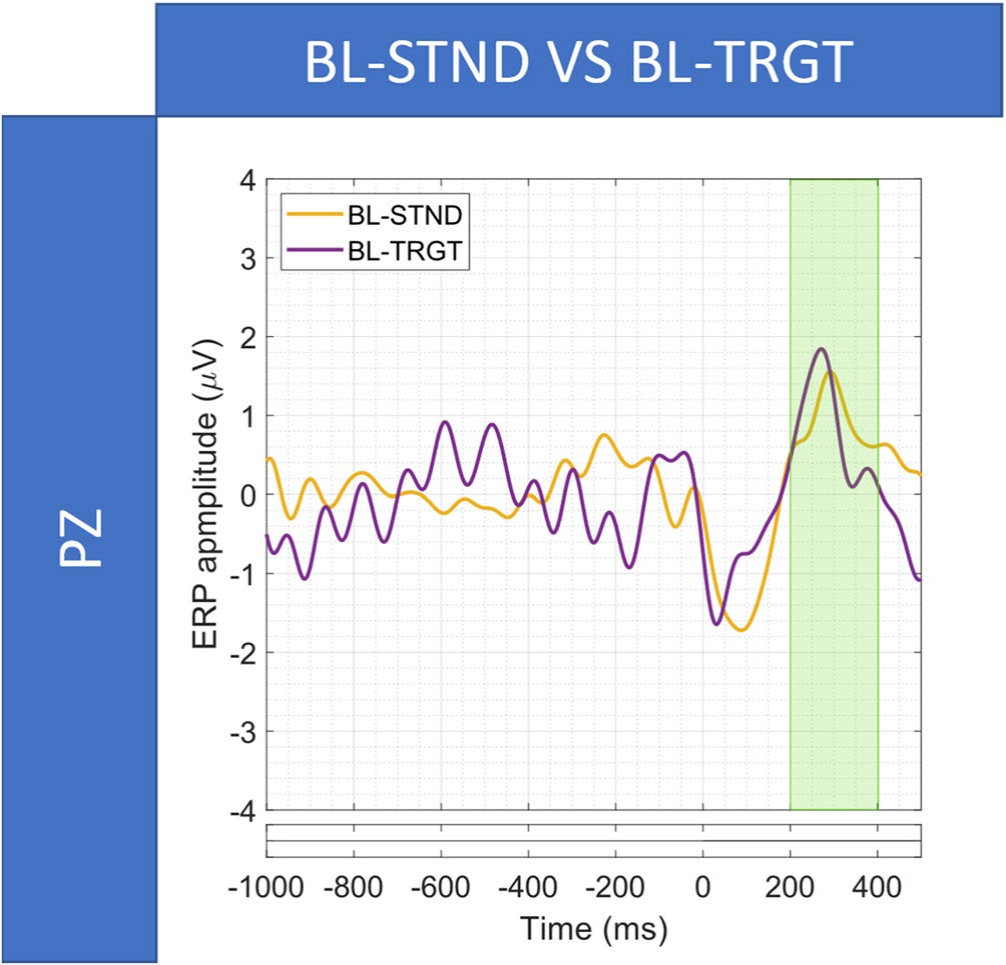
(**a**) BL-STND versus BL-TRGT. ERPs and the corresponding WOI. Bottom row: Statistically significant differences between ERPs (*p* < 0.05, FDR-corrected).

Figure 5 shows the results of the SL-STND versus SL-TRGT (Fig. 5a), SL-STND versus RL-TRGT (Fig. 5b) and the SL-TRGT versus RL-TGRT (Fig. 5c) comparisons. For each analysis, we report the ERP waveforms, along with the temporal range in which we observed significant differences between ERP amplitudes (*p* < 0.05, FDR-corrected). Figures 6 and 7 show the results for SL-STND versus BL-STND (Fig. 6a) and SL-TRGT versus BL-TRGT (Fig. 6b), and for BL-STND versus BL-TRGT (Fig. 7), respectively.

Our findings confirmed the results of^59^, even if we conducted the present study by means of different analysis methods.

Moreover, the presence of the BRP components (associated with both the relevant and irrelevant stimuli) was demonstrated within the windows of interest only when epochs were time-locked to the blink-events (blink-locked), whereas mostly failed to be detected when epochs were aligned both to the stimuli (stimulus-locked) and the motor responses (response-locked). This result confirms BRPs as true neuronal correlates of blink. In fact, stimulus- and response-locking are probably not sufficient to substantially reduce the neuronal jitter due to the variance of both responses to stimuli and behavioural motor responses, causing the dispersion of the BRP waveform on the temporal axis and, thus, the lack of statistically significant responses within the expected window (but rather immediately outside of it) (Fig. 5a,b). On the contrary, only the blink-locked alignment, by eliminating the variance of blinks, probably is able to ensure the proper temporal summation of the BRP and, thus, the adequate synchronization of neuronal responses so as BRP can significantly emerge from the background noise.

Furthermore, the blink-locked BRP components showed similar amplitude for both blinks associated with a target stimulus and blinks associated with a standard stimulus demonstrating that they are not affected by the cognitive load of the stimulus..

In the following, the results of each comparison are reported in detail:

1. *SL-STND versus SL-TRGT.* A statistically significant difference between target and standard response was centered in the 300–500 ms WOI, confirming the results of^59^ and being coherent with the well-known characteristics of the P300^54^. Statistically significant differences were also detected in the 800–1000 ms WOI for STND BRP, but not in the 1000–1200 ms WOI for TRGT BRP (Fig. 5a).
2. *SL-STND versus RL-TRGT.* The amplitude of the P300 in response to STND stimuli was significantly lower than that elicited by the button press after the TRGT stimuli. No other differences were found in the WOIs for the motor response component of the P300 (i.e., 400–600 ms), for the STND BRP (800–1000 ms), and for the response BRP (1100–1300 ms) (Fig. 5b).
3. *SL-TRGT versus RL-TRGT.* Although an evident peak coherent with both the expected motor response components of the P300^72^ and the results reported by^59^ was clearly visible at about 550 ms, no significant difference was found in the 400–600 ms WOI (Fig. 5c). Likewise, we did not observe any significant difference in the 1100–1300 ms WOI for response BRP.
4. *SL-STND versus BL-STND.* A statistically significant difference (target vs standard) was detected within the 800–1000 ms WOI for STND BRP (Fig. 6a).
5. *SL-TRGT versus BL-TRGT.* A statistically significant difference was found in the 1000–1200 ms WOI for TRGT BRP (Fig. 6b). Note from Fig. 6b that a statistical significance difference was also detected in the time window spanning approximately from 640 to 700 ms after stimulus target (that is to say, from 140 to 80 ms before blink).
6. *BL-STND versus BL-TRGT.* No differences were found between TRGT versus STND in the expected time window for BRP (200-400 ms), as shown in Fig. 7).

## Discussion

In this work, we showed that the classical visual oddball task modulates blinking behaviour and its neuronal correlates. Analyses on ERBs indicated that, although the average blink frequency did not deviate from its value at rest^1^, blink latency seemed to be driven by the task regardless of stimulus relevance (i.e., STND vs TRGT). Moreover, we observed that for a set of brain sources located in the precuneus and used to reconstruct the signal on the scalp, differences in BRPs did not depend on stimulus relevance, promoting the idea of their involvement in primary consciousness phenomena. Furthermore, we showed BRPs components even during the pre-blink interval confirming the presence of blink-related neural processing just before the blink onset^75^. Finally, for the same sources, we confirmed the well-known differences in P300 between standard and target stimuli^59^.

### Blink delay after behavioural response

Albeit occurring in response to only 46% of standard stimuli and 48% of target stimuli, blinks following targets occur later than those following standards. Thus, the type of stimulus seems also be able to affect the temporal latency of the subsequent blink. Nevertheless, if we measure the blink latency starting from the button press rather than from the target stimulus administration, this is comparable to the latency of the blink following the standard stimulus. Consequently, the latency of the blink from what we can consider the last meaningful event within the experimental context, be it the delivery of the standard stimulus or the motor response to the target stimulus, remains substantially unchanged. What changes is the duration of the stimulus processing required by the task. The standard stimulus, not being relevant for the task and not requiring a motor response, is processed faster than the target stimulus: with the decision-making choice not to respond to the irrelevant stimulus (NoGo), the processing is virtually concluded. On the contrary, with the decision-making choice to respond to the target stimulus (Go), which is relevant to the task, the motor response must necessarily follow so that the task can be achieved, and the processing phase concluded. As a consequence, the blink in response to the target stimulus is emitted later than that in response to the standard. Therefore, it appears clear that the need to include a motor response as part of the task, expanding both attention and processing times, delays the emission of the blink.

### Blink timing in relation to stimulus timing

Let us imagine a scenario in which two time-series, the sequence of spontaneous blinks at rest and the sequence of external stimuli during the task, each with its own rhythm, interact with each other. On the basis of previous studies^13,14^, the former series can be considered an expression of the internal mental activity not related to the task and therefore connected to the activation of the DMN (endogenous series). The latter series requiring a behavioral response to stimuli, whether overt (i.e., a motor response execution) or covert (i.e., motor response inhibition), certainly involves the activation of the TPN (exogenous series). We speculate that the latter series, due to its behavioural relevance, could behave as a sort of blink attractor, consequently modifying the temporal density of blinks around the stimuli themselves. This seems to be supported by some recent evidence. In fact, it has been demonstrated that humans learn to strategically adapt their blinking behaviour to environmental regularities to better detect future events^76^. Thus, most of the blinks tend to concentrate in the area of low event probability, whereas only a few of them are emitted in the area of high event probability^76^. Furthermore, Rac-Lubashevsky and collegues^23^ have shown that both the update mode (gate opening) and the maintenance mode (gate closing) of a WM reference-back task are followed within seconds by an increase in the event-based eyeblink rate, as an expression of a DA phasic response. This increase occurs either before or after the stimulus depending on the context: if the sequence of stimuli is highly predictable, this blinking adaptation mechanism may be mainly anticipatory (i.e., proactive, or context-driven), whereas if highly unpredictable it may be mainly postpositive (i.e., retroactive, or stimulus-driven)^23^. Thus, since in the present study the cadence of the stimuli was highly unpredictable, it is well understood how blinks occurred after stimuli themselves.

Hence, the sequence of endogenous blinks appears to serve some essential function that cannot be interrupted but, if anything, only modulated by environmental demands. Until now, a certain amount of experimental evidence has been accumulated which leads us to consider this function as a form of nuclear consciousness which relates each other the individual's internal and external environments^13,14^. Following this reasoning, we speculate that blinking behaviour during a visual oddball task could be meant as the result of a trade-off between the need to segment information coming from the external environment and the need to segment information pertaining to one’s own inner environment.

### Lack of a true 1:1 correspondence between stimuli and blinks

Let us assume that such a correspondence does exist and take into consideration the two extreme cases. If the stimuli were very frequent, not only frequent blink-dependent interruptions of vision would occur (with the consequence of increasing the overall time spent with closed eyes), but also attentional blink phenomena^77^, with the result to achieve a high probability of losing a substantial part of the incoming visual stimuli (and thus would cause a decline in performance due to stimulus overflow). Conversely, if the stimuli were exceedingly rare, staying too long waiting for the single stimulus without blinking (and therefore losing the blink-dependent effect of refreshing attention, see later) would lead to time-dependent decay of the outward attention (with consequent decline in performance due to environmental detachment).

Therefore, blinking in response to a stimulus is not an obligatory event but rather an opportunistic one. However, the overall average blinking rate should remain within threshold limits within which the correspondence between stimuli and blink (and therefore the balance between TPN and DMN) reaches an optimal equilibrium (inverted U curve): outside these limits (above and below) such a correspondence would tend to become weaker and vanish.

### Relationship between blinks and P300

The grand-average event-related blink (ERB) peaks after the P300, therefore after the closure of the stimulus processing, whether implicit (to the irrelevant stimulus) or explicit (to the relevant one). This would place the blink in later time windows than response processing, that are more suited to response evaluation phenomena and, consequently, more consistent with the role of environmental monitoring that has been attributed to blink-related potentials (BRPs)^13,14^ and the corresponding blink-related activation of the precuneus^41,42,73^. Phenomena of self-evaluation of the behavioural response has been recently associated to the precuneus activation^59^, establishing an interesting link, albeit indirect, with the role of blinking in the reward-driven task performance and the visuomotor binding^2^.

### BRP amplitude does not depend on the stimulus cognitive valence

The fact that the amplitude of BRPs following relevant stimuli is not significantly different from that of irrelevant stimuli, unlike what happens for P300, suggests that (1) blink-related responses are not influenced by the cognitive valence of the stimulus (i.e., by the stimulus intrinsic cognitive weight); and thus (2) blink-related responses could be generated by more basic phenomena such as primary consciousness phenomena, which by their nature are considered all-or-nothing, not modulable, phenomena^78^. In fact, primary consciousness phenomena must accompany the perception and processing of the relevant stimulus as well as of the irrelevant one so that the task can be effectively accomplished: in several real-life situations, in fact, not providing a motor response to a stimulus^79^ constitutes an actual (even implicit, covert) behavioural response.

In this regard, it is interesting to note that the precuneus has also been implicated in early cognitive processes such as the encoding of distractors and the inhibition of their subsequent processing^80,81^, being able to influence early stages of both visual^10,81^ and auditory^82^ perceptions.

### Pre-blink components of BRPs

We observed the presence of blink-related neural processing even during the pre-blink interval confirming recent findings of Liu et al.^75^. Pre-blink components of BRPs have been related to the brain's need to prepare itself to process the incoming blink-related visual information in the face of the ongoing visual input^75^. Such a competition requires to be resolved through dynamic adaptation processes such as an anticipatory modulation of the blink-related processes^75^. The importance of these processes is further corroborated if blink is considered as an element of connection and transition between contiguous attentional spans, as will be discussed in the next paragraph.

### Neural correlates of blinking and consciousness phenomena

The present results suggest that BRPs during goal-oriented behaviour may deal with ongoing primary consciousness phenomena related to the contextual situation, putatively including in this term one’s own inner contextual environment. BRPs have been recently showed (and also confirmed in the present study) not only during the attentional span which follows the blink but also during the immediately preceding one^75^. In this perspective, we hypothesize that the blink could represent the moment in which both updating and short-term storage occur of that contextual information^13,14^ that will serve as a substrate for the processing of the subsequent span, in a continuously ongoing process of comparison and updating between just past and present experiences -the “remembered present” by Edelman^83^. In this way, the blink would assume a role of connection between consecutive segments of processing in which focused attention is disengaged from the external environment (i.e., the task) to be momentarily oriented towards the internal environment and viceversa^37,43^. This interplay between outward- and inward-directed attention, between self and out-of-self, is intriguingly the same that Crick and Koch referred to as the very mechanism of consciousness^84^. Moreover, it is important to note how low frequency oscillations of blink-related responses are missing in patients affected with unresponsive wakefulness syndrome in which consciousness is totally lacking^41,58^. We speculate that such a flip of attentional focus orientation during blink may be necessary for maintaining the effectiveness and efficiency of TPN during goal-oriented behaviour. In fact, outward attention (and its underlying functional network, TPN) may need to transiently detach from its object to refresh and be able to focus again with renewed strength. Momentarily disconnecting from the task, even totally fleeting, would have the purpose of counteracting the temporal decay of outward attention due to habituation phenomena^85,86^, thus allowing outward attention to re-engage the task with restoring force.

This hypothesis is consistent with the suggestion that the precuneus may play a role in modulating selective attention for optimal allocation of resources^59^. Interestingly, it has been recently shown from several fMRI studies that the precuneus, far from being shut down during cognitive tasks, comes dynamically into play together with the TPN, working as a transient in-between hub connecting the DMN to task-positive areas with a context-dependent modulatory behaviour^87^ and playing for example a putative role in the background exploration and monitoring of alternative courses of action^88^. In the light of the results of the present work, it might be interesting to investigate in the future whether precuneal fMRI activations during cognitive tasks are due to spontaneous blinking.

In this vein, blinking could become a moment of transition (or resetting) between an attentional phase that has faded out and a renewed, refreshed attentional phase. This scenario is compatible with the idea proposed by Holland and Tarlow^32^ that blinking is the expression of a cognitive or brain state change. A concept that, as already stated above, is linked per se to the blink^13,14^ and can be considered similar to those of updating and gate switching (even if during the present task rules did not strictly change) by which blinking is known to be driven^2^.

Finally, we hypothesize that the blink closes an attentional span, be it outward or inward directed, and opens the next one, contextually enabling a brief transient activation of the DMN. Blink-related potentials as well as the corresponding precuneus activation could then represent the neural correlates of those primary consciousness phenomena that lie at the interface of the transition between DMN and TPN.

### Limitations

In the present study, the resting control condition is missing as well as in Walz’s study from which the dataset we analyzed was obtained. Thus, we did not have the opportunity to investigate whether a difference exists in both blink rate and BRPs between rest and task conditions so as to shad lights on two complementary aspects of blinking behaviour.

A limitation of the current study derives from the rule used to extract blinks. Particularly, by considering only the first blink in the (0,1500)ms time window following stimulus administration, we are excluding multiple blinks which it may be associated with neural activity (i.e., BRPs). In this light, although the presence of multiple blinks within an epoch was limited (< 5% of times), their effect would be that of introducing noise in the analysis. Here, we accepted such a level of noise as negligible because of the limited number of blinks of this type. However, other studies could consider the activity arising from multiple blinks in the analysis.

Another potential limitation of the study concerns the possible presence of non-spontaneous blinking. However, since subjects did not have any specific instruction on promoting/limiting blinks^59^, although we cannot exclude the presence of non-spontaneous blinks, we can reasonably assume that they are negligible for our analysis.

Finally, it is worthwhile noting that some blinks occur at relatively large latencies. In this light, including these blinks in the grand average BRP inevitably introduces a certain amount of noise in the analysis, as they may be related to other processes not related to the task. However, we can reasonably assume that given the low proportion of such blinks, they do not affect the final outcome. In this light, other strategies could be adopted, for instance, reducing the window for including blinks in the analysis.

## Conclusion

The present results show that (1) the rhythm of blinking during a visual oddball task is modulated by stimuli, whether they are relevant or irrelevant to the task; (2) blinks are delayed by behavioural responses; and (3) the stimulus relevance does not influence the magnitude of blink-related potentials as detected from precuneal clusters. This suggests a role of blinking in pacing rhythms of cognition and its involvement in contextual primary consciousness processes.

## Data Availability

The data used in this work is freely available at: https://legacy.openfmri.org/dataset/ds000116/.
